# Monitoring physical and psychosocial symptom trajectories in ovarian cancer patients receiving chemotherapy

**DOI:** 10.1186/1471-2407-12-77

**Published:** 2012-02-28

**Authors:** Verena Meraner, Eva-Maria Gamper, Anna Grahmann, Johannes M Giesinger, Petra Wiesbauer, Monika Sztankay, Alain G Zeimet, Barbara Sperner-Unterweger, Bernhard Holzner

**Affiliations:** 1 Department of Psychiatry and Psychotherapy, Innsbruck Medical University, Anichstr. 35, A-6020 Innsbruck, Austria; 2 Rosenegg 48a, A-6391 Fieberbrunn, Austria; 3 Department of Gynecology and Obstetrics, Innsbruck Medical University, Anichstr. 35, A-6020 Innsbruck, Austria

## Abstract

**Background:**

Diagnosis and treatment of ovarian cancer (OC) entail severe symptom burden and a significant loss of quality of life (QOL). Somatic and psychological impairments may persist well beyond active therapy. Although essential for optimal symptom management as well as for the interpretation of treatment outcomes, knowledge on the course of QOL-related issues is scarce. This study aimed at assessing the course of depressive symptoms, anxiety, fatigue and QOL in patients with OC over the course of chemotherapy until early after-care.

**Methods:**

23 patients were assessed longitudinally (eight time points) with regard to symptom burden (depression, anxiety, fatigue, and QOL) by means of patient-reported outcome instruments (HADS, MFI-20, EORTC QLQ-C30/-OV28) and clinician ratings (HAMA/D) at each chemotherapy cycle and at the first two aftercare visits.

**Results:**

Statistically significant decrease over time was found for depressive symptoms and anxiety as well as for all fatigue scales. With regard to QOL, results indicated significant increase for 11 of 15 QOL scales, best for Social (effect size = 1.95; *p *< 0.001), Emotional (e.s. = 1.62; *p *< 0.001) and Physical Functioning (e.s. = 1.47; *p *< 0.001). Abdominal Symptoms (e.s. = 1.01; *p *= 0.009) decreased, Attitudes towards Disease and Treatment (e.s. = 1.80; *p *< 0.001) improved significantly over time. Analysis of Sexual Functioning was not possible due to a high percentage of missing responses (61.9%).

**Conclusions:**

The present study underlines the importance of longitudinal assessment of QOL in order to facilitate the identification of symptom burden in OC patients. We found that patients show high levels of fatigue, anxiety and depressive symptoms and severely impaired QOL post-surgery (i.e. at start of chemotherapy) but condition improves considerably throughout chemotherapy reaching nearly general population symptoms levels until aftercare.

## Background

According to GLOBOCAN estimates [1], 224.747 women worldwide were diagnosed with ovarian cancer (OC) in 2008. Fatally enough, early stage disease usually presents with minor and unspecific symptoms [2,3], considerably prolonging the time period between occurrence of first complaints and physician consultation. This circumstance is the major reason why in 75% of the concerned patients OC is diagnosed at an advanced stage (i.e. FIGO stages III and IV) [4] and prognosis is consequently poor. Therefore, OC is called the "silent killer". In the majority of cases, treatment involves cytoreductive surgery with obligatory removal of the uterus and both adnexa as well as post-operative chemotherapy. The chemotherapeutic approach usually consists of a platinum-taxane combination [5] administered either intravenously or directly into the peritoneal cavity [6]. Despite this intensive, burdensome treatment, patients still have to face a considerable risk of recurrence [7].

Advances in the treatment of OC, however, contribute to increased survival rates. Thus, quality of life (QOL) issues are gaining importance for patients and caregivers extending treatment goals from mere prolongation of disease-free periods to maintenance of functioning and well-being. The latter proves challenging in the light of knowledge about toxicity. OC patients have to deal with a range of treatment-related symptoms such as hematological side-effects, gastrointestinal problems, neuropathic pain, menstrual changes together with climacteric symptoms, and fertility issues [8-10]. Furthermore, the majority of OC patients experiences fatigue [8,11,12] severely affecting patients' daily lives far beyond the completion of treatment [13]. Accordingly, it was ranked as the most important symptom by both, patients [14] and physicians [15].

Other problems, although highly prevalent, are less communicated during physician consultation. This applies to a range of QOL issues such as sexual [16] and emotional difficulties [17]. Routinely conducted monitoring of symptom burden considerably contributes to the detection as well as to the systematic investigation of neglected symptoms and aspects of functioning. Several studies in this field found OC patients to experience high levels of distress over delayed diagnoses as well as anxiety and depression [9,18-21], both good predictors for global QOL [12]. The role of QOL itself as a predictor of disease outcome and survival in OC patients is yet controversially discussed [18,22]. Nonetheless, QOL proves essential not only for optimal symptom management but also as additional information in the evaluation of treatment outcomes in clinical trials. Knowledge, however, on the course of QOL issues during therapy is scarce in OC patients.

Therefore, this study was designed to assess a wide range of disease- and treatment-related issues relevant to OC patients from the beginning of chemotherapy until early aftercare in a longitudinal approach. Thus, it addressed the trajectory of depressive symptoms, anxiety, fatigue and QOL in patients with OC.

## Methods

### Sample

OC patients at the Department of Gynecology and Obstetrics at Innsbruck Medical University were included in the study at the beginning of adjuvant intravenous chemotherapy.

Additional inclusion criteria comprised: age between 18 and 85 years, expected survival time of at least 3 months, no overt cognitive impairments, fluency in German language as well as written informed consent.

### Procedure

Patient recruitment started in 2003 and data assessment was completed in 2006. Eligible patients were approached by a physician or a psycho-oncologist at their routine appointments for chemotherapy administration and were consecutively included.

Assessments concerning depressive symptoms, anxiety and fatigue were completed at each chemotherapy cycle and at the first two aftercare visits (i.e. three and 6 months after termination of the chemotherapy, respectively). QOL, subsuming physical as well as psychosocial symptoms, was assessed at the first, the third as well as the sixth chemotherapy cycle and at both aftercare visits. Baseline data was collected post-surgery at first chemotherapy cycle.

Overall, it was expected to cover a time frame of about 9 months in each patient. Ethical approval for this project was obtained from the Ethics Committee of Innsbruck Medical University.

### Assessment instruments

Sociodemographic variables included age, education, employment and marital status. Clinical data comprised FIGO stage, histological subtype, presence of residual disease after primary debulking surgery, number of total chemotherapy cycles and menopausal status. Data was gathered from hospital records.

To evaluate progression of physical and psychosocial symptoms over the course of chemotherapy, we applied the following assessment instruments.

#### Hospital Anxiety and Depression Scale (HADS)

The Hospital Anxiety and Depression Scale (HADS) [23] is a widely used, validated screening instrument for anxiety and depressive symptoms in somatically ill patients. It is a short self-assessment scale comprising 14 questions, addressing anxiety and depressive symptoms with 7 items each at the time-frame of the previous 7 days.

#### Hamilton Anxiety and Depression Scales (HAMA, HAMD)

The Hamilton Anxiety as well as Depression Scales are semi-structured interviews developed for the clinical evaluation of severity of anxiety and depression in adults.

The HAMA [24] consists of 14 items which are defined by a series of symptoms measuring both psychic anxiety (mental agitation and psychological distress) and somatic anxiety (physical complaints related to anxiety). The HAMD [25] is a 21-item scale evaluating depressed mood, vegetative and cognitive symptoms of depression and comorbid anxiety symptoms. It provides ratings on current DSM-IV symptoms of depression, with the exceptions of hypersomnia, increased appetite and concentration/indecision.

In both interviews items are rated on a five-point Likert scale, higher total scores indicating more severe anxiety or depression.

#### Multidimensional Fatigue Inventory (MFI-20)

The MFI-20 [26] is a 20-item self-report instrument designed to measure fatigue by means of the five subscales General Fatigue, Physical Fatigue, Mental Fatigue, Reduced Motivation, and Reduced Activity. Each subscale comprises four items that are rated on a five-point Likert scale, higher scores indicating a higher level of fatigue.

#### EORTC Quality of Life Questionnaires (QLQ-C30, QLQ-OV28)

The EORTC QLQ-C30 [27], an internationally validated and widely used cancer-specific QOL-instrument, assesses various facets of functioning and symptoms common in cancer patients. It comprises five functioning scales (physical, social, role, emotional, cognitive), a scale for global QOL, and nine symptom scales (fatigue, nausea/vomiting, pain, dyspnea, sleeping disturbances, appetite-loss, constipation, diarrhea, and financial impact). All scales are scored according to EORTC guidelines resulting in a score range from 0 to 100 points.

The QLQ-OV28 module [28] is a supplement of the QLQ-C30 for assessing issues relevant to ovarian cancer patients. The module covers abdominal/gastrointestinal symptoms, peripheral neuropathy, other chemotherapy side-effects, hormonal/menopausal symptoms, body image, attitude to disease/treatment and sexual functioning.

With reference to Osoba et al. [29], differences in QOL-scores of 10 points or more should were considered as moderate and differences of 5 to 10 points as small.

### Statistical analysis

Sample characteristics are presented as frequencies, percentages, means, standard deviations and ranges.

Longitudinal analyses of the outcome measures were conducted using mixed linear models, including fixed effects and random intercepts on patient level. The sequential numbers of the assessment time points (0 to 7) were included in the model as covariate. Although, especially with regard to aftercare, time points were not equidistant, this was considered reasonable to model the slightly quadratic shape of the regression curve, indicating slower recovery in aftercare. This quadratic effect was expected since patients' condition improved much until the end of chemotherapy, so that further recovery was likely to occur in greater intervals. More complex statistical modeling was not appropriate due to sample size. Regression weights (beta) are given for significant fixed effects.

Effect sizes (Cohen's d) were calculated as difference between baseline and last assessment divided by baseline standard deviation.

## Results

### Patient characteristics

Due to administrative reasons patients could only be approached randomly. Twenty three patients (30% of eligible patients treated at the Department of Obstetrics and Gynaecology at Innsbruck Medical University during the study period) could be recruited at beginning of chemotherapy. Two patients withdrew consent (due to expected burden and language difficulties) and were excluded from statistical analysis. No patient has been lost to follow-up. Mean patient age was 52.8 years (SD 13.1). The most frequent histopathologic cancer type was serous carcinoma (66.7%). The most common FIGO stage was III (76.2%). 20 patients were treated with a carboplatine-paclitaxel combination and one patient received cisplatin/etoposid. 17 patients received 6, one patient 7 and three patients 9 cycles of first line chemotherapy. All patients were assessed at the first six cycles and subsequently, in aftercare. One patient was diagnosed with recurrent disease during early aftercare. 15 patients (71.4%) received psycho-oncological counseling at some point during the study (6 patients > 10 visits, 5 patients 5-10 visits, 4 patients < 5 visits). Further details on patient characteristics are summarized in Table 1.

**Table 1:** Descriptive statistics for sociodemographic and clinical variables of the 21 eligible patients with ovarian cancer at the time of study inclusion before the initiation of adjuvant chemotherapy

			n	%
**Age [in years]**	***mean (SD)*** ***range***	**52.8 (13.1)** **27-74**		

Education	compulsory school or less		15	**71%**
	apprenticeship/professional		2	**10%**
	school			
	A-level/university		4	**19%**

Marital status	single		1	**5%**
	married/with partner		16	**75%**
	divorced/separated		2	**10%**
	widowed		2	**10%**

Employment status	full employment		4	**19%**
	part-time employment		6	**29%**
	homemaker		6	**29%**
	retired/pension		4	**19%**
	other		1	**5%**

Menopausal status	pre-menopausal		9	**43%**
	post-menopausal		12	**57%**

FIGO stage	I-II		6	**27%**
	III-IV		15	**73%**

Histological subtype	mucinoid carcinoma		3	**14%**
	serous carcinoma		16	**76%**
	endometrioid carcinoma		1	**5%**
	other		2	**10%**

Residual disease	none		7	**33%**
	yes		12	**57%**
	missing		2	**10%**

Chemotherapy regimen	carboplatin/paclitaxel		20	**95%**
	cisplatin/etoposide		1	**5%**

Chemotherapy cycles	6		17	**81%**
	7		1	**5%**
	9		3	**14%**

### Time course of depressive symptoms and anxiety

**Figure 1:**
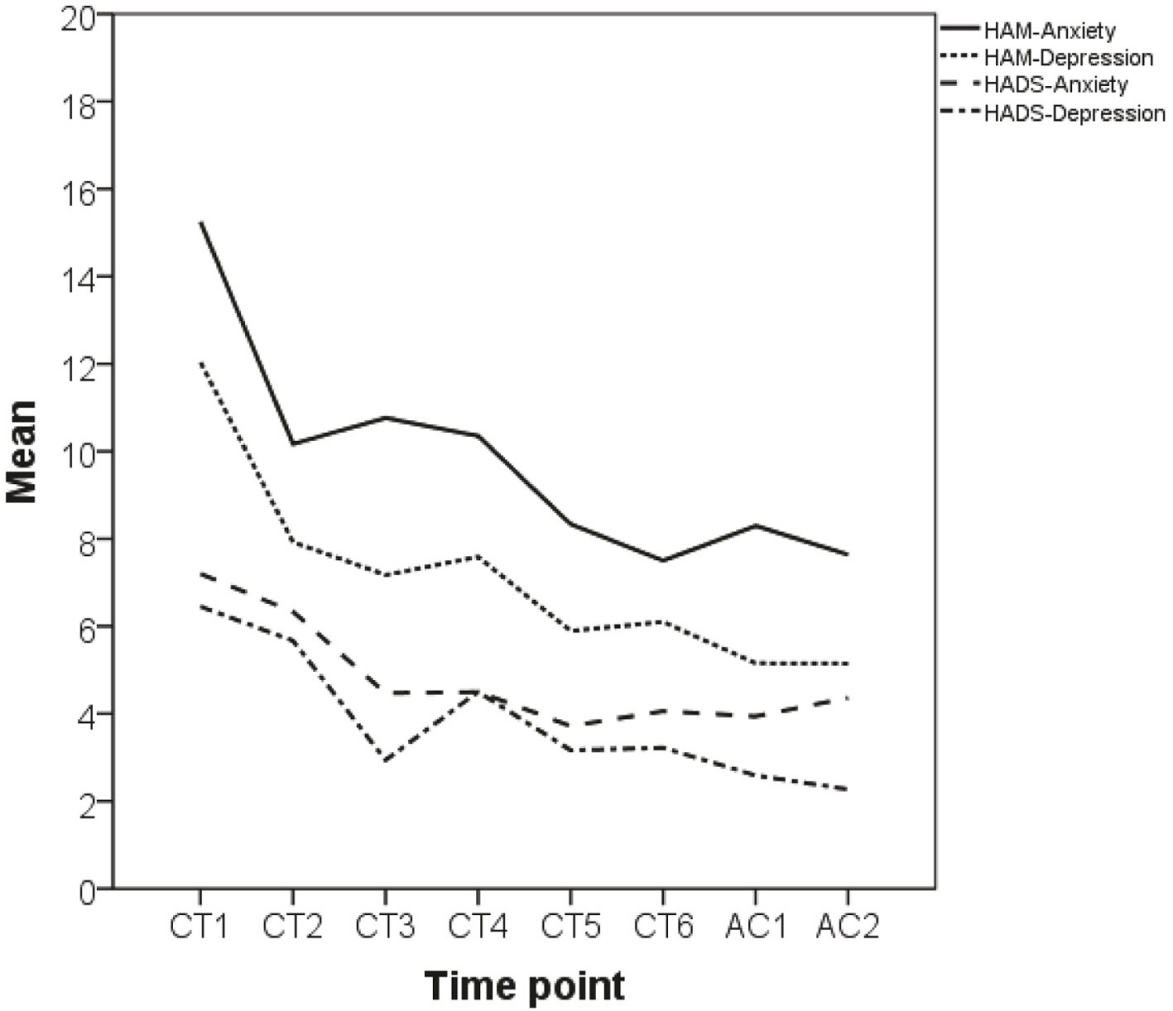
**Time course of anxiety and depressive symptoms in the 21 patients with ovarian cancer as assessed with the Hospital Anxiety and Depression Scale (HADS) and the Hamilton Anxiety and Depression Scale (HAM)**.

Assessment via self-reports (HADS) as well as via expert-ratings (HAMA/D) at each chemotherapy cycle and at the first two aftercare visits revealed a significant decrease over time for both, the prevalence of depressive symptoms and anxiety. Effect size was 1.11 for HADS-Depression, 1.03 for HAM-Depression, 0.93 for HAM-Anxiety and 0.69 for HADS-Anxiety. For further details see Table 2 and Figure 1.

**Table 2:** Course of Fatigue, Anxiety and Depression as assessed in 21 ovarian cancer patients with three different assessment instruments

	BL^a^	CT2^b^	CT3	CT4	CT5	CT6	**AC1**^b^	AC2	Constant Term/ Beta Coefficient		Reference Values^c^
**Hospital Anxiety and Depression Scale (HADS)**^c^											

Anxiety	7.0 (4.2)	6.1	4.5	4.5	3.9	4.1	4.5	4.1 (4.3)	5.7/-0.3	t = -2.80; *p *= 0.006	**5.0** **(3.4)**

Depression	6.5 (3.8)	5.4	3	4.5	3.5	3.3	3.1	2.3 (3.6)	5.2/-0.3	t = -4.07; *p *< 0.001	**4.7** **(3.9)**

**Hamilton Anxiety and Depression Scale (HAMA/D)**											

Anxiety	15.0 (8.0)	11.0	11.4	10.4	8.3	7.5	9.2	7.6 (5.9)	12.8/-0.6	t = -3.01; *p *= 0.004	**n.a**.

Depression	11.7 (6.8)	8.1	7.9	7.6	5.6	6.6	5.2	4.7 (3.2)	9.3/-0.6	t = -3.58; *p *= 0.001	**n.a**.

**Multidimensional Fatigue Inventory (MFI)^c^**											

General Fatigue	13.2 (4.7)	13.4	11.9	12.8	11.8	11.7	9.7	7.8 (3.9)	13.4/-0.5	t = -4.62; *p *< 0.001	**10.8** **(3.7)**

Physical Fatigue	13.1 (3.9)	13.5	13.4	12.9	11.2	11.2	9.6	7.4 (4.4)	13.7/-0.5	t = -5.29; *p *< 0.001	**11.1** **(4.2)**

Reduced Activity	15.4 (4.8)	15.1	12.2	12.4	10.9	11.2	8.7	6.5 (3.6)	15.2/-1.0	t = -8.19; *p *< 0.001	**10.5** **(4.0)**

Reduced Motivation	7.5 (3.5)	7.4	6.5	7.9	6.4	6.3	6.2	5.6 (1.9)	7.5/-0.2	t = -2.98; *p *= 0.004	**9.9** **(3.5)**

Mental Fatigue	9.8 (5.1)	8.6	8.8	9.6	8.2	7.7	7.5	7.3 (4.3)	9.3/-0.2	t = -2.03; *p *= 0.046	**9.2** **(3.4)**

^a ^BL baseline (CT1)

**^b ^**CT chemotherapy cycle, AC aftercare

**^c ^**Reference values: *HADS *(female general population) [31]*MFI-20 *(general population > 60 y) [30]

### Time course of fatigue

**Figure 2:**
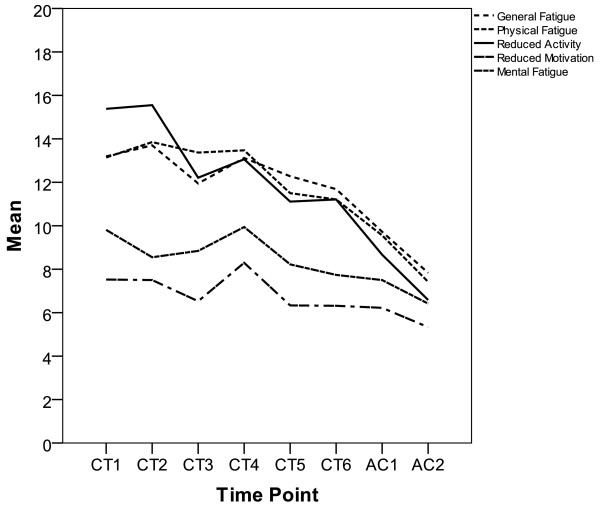
**Time course of various aspects of fatigue in the 21 patients with ovarian cancer as assessed with the Multidimensional Fatigue Inventory (MFI-20)**.

A statistically significant decrease over the course of treatment was found for all MFI scales. The decrease in terms of baseline standard deviation units was strongest for Reduced activity (e.s. = 1.85), Physical Fatigue (e.s. = 1.46) and General Fatigue (e.s. = 1.15). Lower but still significant was the change in Reduced Motivation (e.s. = 0.54) and Mental Fatigue (e.s. = 0.47). Similarly, the Fatigue scale of the EORTC QLQ-C30 indicated a strong improvement over time (e.s. = 1.77). For further details see Tables 3 and 4 as well as Figure 2 depicting the time course.

**Table 3:** Course of Quality of Life as assessed in 21 ovarian cancer patients with the EORTC Quality of Life core questionnaire (QLQ-C30)

EORTC QLQ-C30^c^							
	**Baseline^a^**	**CT3^b^**	**CT6**	**AC1**^b^	**AC2**	**constant term/** **beta coefficient**	

Physical Functioning (PF)	53.7 (27.3)	62.0	65.9	78.5	93.9 (8.7)	50.1/5.9	t = 7.31; *p *< 0.001

Social Functioning (SF)	34.1 (26.6)	53.9	63.7	76.5	86.1 (15.6)	34.3/7.2	t = 7.29; *p *< 0.001

Role Functioning (RF)	40.5 (35.2)	36.3	47.1	62.0	77.8 (27.8)	32.0/5.0	t = 3.74; *p *< 0.001

Emotional Functioning (EF)	47.6 (24.6)	64.2	69.6	79.9	87.5 (15.7)	49.0/5.3	t = 5.93; *p *< 0.001

Cognitive Functioning (CF)	77.0 (25.5)	73.5	86.3	86.3	90.3 (16.6)	75.0/2.0	t = 2.22; *p *= 0.031

Global QOL (GQOL)	47.5 (14.3)	56.4	59.8	58.3	60.4 (29.3)	49.0/1.9	t = 2.405; *p *= 0.019

Fatigue (FA)	66.1 (28.2)	51.6	51.6	33.3	16.2 (16.8)	67.2/-6.3	t = -6.18; *p *< 0.001

Nausea/Vomiting (NV)	23.8 (30.1)	15.6	14.7	2.8	5.6 (13.0)	23.1/-3.0	t = -3.35; *p *= 0.002

Pain (PA)	46.0 (37.6)	35.3	33.3	25.9	10.6 (13.5)	47.9/-4.8	t = -4.18; *p *< 0.001

Dyspnoe (DY)	19.0 (34.3)	13.7	23.5	7.4	8.3 (20.7)	18.5/-1.5	t = -1.36; *p *= 0.178

Sleeping Disturbances (SD)	38.1 (38.4)	35.3	23.5	55.6	13.9 (33.2)	39.5/-3.1	t = -1.91; *p *= 0.062

Appetite Loss (AP)	57.1 (42.4)	5.9	21.6	7.4	0.0 (0.0)	31.0/-4.0	t = -3.04; *p *= 0.006

Constipation (CO)	25.0 (35.7)	15.7	27.5	14.8	6.1 (20.1)	23.9/-2.0	t = -1.60; *p *= 0.116

Diarrhea (DI)	16.7 (29.6)	11.8	8.3	9.3	0.0 (0.0)	12.2/-0.7	t = -1.43; *p *= 0.167

Financial Impact (FI)	19.0 (30.9)	25.5	13.7	5.9	2.8 (9.6)	23.8/-2.9	t = -3.12; *p *= 0.003

^a ^Baseline (CT1)

^b ^CT chemotherapy cycle, AC aftercare

^c ^Reference values (general German population, female) [53]**: **PF = 88.7 (17.5), SF = 90.3 (20.1), RF = 86.6 (23.7), EF = 76.3 (22.2), CF = 90.1 (18.4), GQOL = 69.2 (21.9); FA = 19.5 (23.1), NV = 3.6 (11.4), PA = 17.2 (25.3), DY = 9.1 (21.6), SD = 19.1 (29.0), AP = 6.3 (17.4), CO = 4.3 (14.9), DI = 3.1 (12.6), FI = 6.3 (18.6)

**Table 4:** Course of Symptoms as assessed in 21 ovarian cancer patients with the ovarian cancer-specific module (OV-28) of the EORTC Quality of Life core questionnaire

EORTC QLQ-OV28^c^							
	**Baseline^a^**	**CT 3**^b^	**CT 6**	**AC1**^b^	**AC2**	**constant term/** **beta coefficient**	

Abdominal Symptoms (AS)	41.9 (28.1)	21.8	29.2	20.5	13.5 (21.7)	36.8/-3.0	t = -2.72; *p *= 0.009

Peripheral Neuropathy (PN)	32.8 (26.2)	37.7	39.2	37.7	23.1 (26.1)	35.3/-0.5	t = -0.39; *p *= 0.697

Other Chemotherapy-related Side Effects (CSE)	30.2 (33.8)	29.3	29.8	23.0	1.6 (2.7)	42.4/-5.7	t = -5.97; *p *< 0.001

Hormonal/menopausal Symptoms (HS)	34.1 (35.1)	22.2	26.5	31.5	22.2 (31.2)	30.2/-0.8	t = -0.55; *p *= 0.587

Body Image (BI)	73.0 (33.1)	74.5	84.3	75.0	77.8 (25.0)	74.2/0.8	t = 0.68; *p *= 0.502

Attitudes towards Disease and Treatment (AT)	26.1 (22.6)	39.5	56.9	53.7	66.7 (24.6)	27.1/5.4	t = 5.30; *p *< 0.001

*Sexual Functioning (SF)*	*47.9 (21.9)*	*64.3*	*48.8*	*57.3*	*57.4 (12.8)*	*48.9/1.0*	*n = 8*

^a ^Baseline (CT1)

^b ^CT chemotherapy cycle, AC aftercare

^c ^Reference values (ovarian cancer patients, all stages) [54]: AS = 21.9 (22.1), PN = 17.1 (27.2), CSE = 19.5 (20.5), HS = 25.8 (30.3), BI = 22.2 (27.4), AT = 42.3 (30.6)

### Time course of quality of life and symptoms

With regard to the QLQ-C30, significant increase was found for 11 of 15 Functioning subscales. The improvement was best for Social Functioning (e.s. = 1.95), Emotional Functioning (e.s. = 1.62) and Physical Functioning (e.s. = 1.47). Changes for Role Functioning (e.s. = 1.06), Global QOL (e.s. = 0.90) and Cognitive Functioning (e.s. = 0.52) were lower, but still significant. Furthermore, significant improvements were found for Fatigue (e.s. = 1.77), Pain (e.s. = 0.94), Nausea/Vomiting (e.s. = 0.60) and Financial Impact (e.s. = 0.52).

Since for the scales Appetite Loss and Diarrhea none of patients reported a value above 0 at the second aftercare visit, this time point had to be excluded from the mixed linear model due to lack of variance. Whereas, for Diarrhea no significant time effect was found for the remaining time points, Appetite Loss decreased significantly (e.s. = 1.17).

The Ovarian Cancer Module QLQ-OV28 revealed a significant decrease in Abdominal Symptoms (e.s. = 1.01) and an improvement for Attitudes towards Disease and Treatment (e.s. = 1.80). Analysis of the scale Sexual Functioning was not possible due to a very large number of missing values (61.9%).

## Discussion

Diagnosis and treatment of OC, mostly comprising debulking surgery and subsequent chemotherapy, entail severe symptom burden and a significant loss of patients' quality of life.

Correspondingly, our study results indicate impairments for a wide range of somatic and psychological symptoms at the beginning of chemotherapy. Thus, at baseline, levels of anxiety and the prevalence of depressive symptoms were increased as reflected by self- as well as proxy-ratings. Similarly, fatigue (in terms of general fatigue, physical fatigue and reduced activity) was found to be high. Patients, however, recovered significantly over time up until aftercare. A strong improvement was not only found for anxiety and regarding the prevalence of depressive symptoms but also for almost all aspects of functioning covered by the QLQ-C30 as well as for fatigue (less pronounced for mental fatigue and reduced motivation). Both HADS as well as MFI-20 scores returned toward general population levels [30,31].

From a clinical point of view, this degree of recovery was remarkable and unexpected forasmuch as patient-reported QOL measures correlate with standard toxicity criteria [32] and provide viable information on the impact of treatment-related toxicity on patients' functioning. Other studies, however, report similar pronounced improvements of QOL in OC patients during chemotherapy until one year follow-up as found in this study [33-36].

Unfortunately, the small sample size did not allow for detailed subgroup analyses, which is the major limitation of the study. Thus, it was not possible to investigate if the observed QOL-scores can be attributed to response to treatment. Bezjak et al. [33], however, surmise that the improvement of QOL is not an effect in treatment responders only but can as well be observed in cancer patients with stable disease.

One possible explanation for the improvement of QOL in the course of treatment is the choice of baseline. In studies on QOL in OC patients, assessing baseline scores post-surgically is considered as standard. Von Gruenigen et al. [34], though, suggest low QOL at the beginning of chemotherapy to be an effect of surgery and, in accordance with Bezjak et al. [33], discuss this low post-operative baseline as the potential reason for rapid QOL improvement.

This interpretation might also apply for the present study. Since baseline scores were assessed at beginning of chemotherapy, i.e. approximately only 10 days after surgery, high somatic and psychosocial symptom burden resulting in low QOL was expected due the burdensome circumstances of this specific treatment period. As chemotherapy has to start without delay, patients are usually not discharged from hospital after surgery, thereby counteracting patients' desire to regain strength and activate resources in their familiar surroundings at home.

This fearful anticipation of a probably fatal outcome is mingled with the fear of chemotherapy and its expected side-effects. Furthermore, the impact of surgery on QOL could also be influenced by tumor stage and thus, the extent of the surgical intervention but also by intra- or postoperative complications. In our sample, distribution of tumor stage can be considered as representative for the population of OC patients [1].

Subsequent increase in QOL might partly be due to the actual initiation of adjuvant chemotherapy, which introduces a structured course of events into the patient's clinical daily life. The patient works towards a foreseeable end of treatment, gaining a feeling of security from the well-regulated procedure, which supports adjustment to the current situation. Moreover, OC patients may very well compensate decreased functioning and well-being with increased social support [37].

Other psychological factors may also contribute to the increase of QOL scores in ovarian cancer patients to a level similar to general population norms.

The importance of patients' expectations in the context of evaluation of QOL has already been pointed out by Wan et al. [38] who demonstrated that the discrepancy between what patients expect and what actually occurs with treatment is a significant predictor for every dimension of health-related QL. The expectation of cure or at least, prolongation of survival, which is more pronounced in the active stage of treatment, might play an important role. Doyle et al. [39] found that 65% of the 27 women with advanced ovarian cancer expected chemotherapy to prolong their survival and 42% to cure them. However, negative and unrealistic expectations might also translate into adverse experiences in the course of treatment [40], which underlines the importance of assistance in terms of psychooncological support in order to establish and maintain realistic QOL expectations [38].

When prior expectations are challenged by actual treatment experiences, patients' perspectives might be altered leading to a possible change in the subjective appraisal of QOL. This 'response shift' is defined as a process of change in internal standards of measurement, values and the self-evaluation as well as conceptualization of quality of life over the course of the disease trajectory [41]. This model provides a dynamic feedback loop to explain how quality of life scores can be stabilized despite changes in objective health status [42].

The possibility of response shift should, however, not blur the fact that "toxicity to which the patient has accommodated still is toxicity" [43].

After completion of treatment and within the time frame of aftercare, patients are relieved about having completed a difficult treatment period. Clinical experience suggests that levels of psychological distress probably increase during later aftercare as integration in medical treatment procedures is less intense and the patient is confronted with the fear of recurrence. Results from a study conducted by Mirabeau et al. [44] investigating long-term adjustment and QOL in survivors after a minimum of 3 years without recurrence, support the clinical impression by showing that anxiety when getting the results of CA-125 relapse-screening is still an issue for these patients. At a 10-year follow up assessment, findings by Greimel et al. [22] showed QOL in OC survivors returning to normal levels, yet in a small sample only.

Accordingly, one other limitation of the present study is that follow-up assessment was terminated after 9 months. Continuous assessment beyond early aftercare might have provided important information on the stability of the rather good condition at study end point. Available literature on long-term quality of life in OC patients, especially in aftercare, is scarce [45] also because long-term survival rates are rather low.

In addition to distress due to fear of recurrence, OC survivors often report sexual problems attributed to cancer [44,46,47]. Unfortunately in the present investigation, no analysis of sexual functioning and impairment was possible given that only a few patients answered the questions pertaining to this matter. This is, however, a common difficulty reported in several studies [28,44].

The problem of some issues not being volunteered by patients is restricted neither to sexual issues nor to questionnaire assessment. As already mentioned previously, even the most prevalent symptoms often remain unaddressed during physician consultation. As a result, only a certain part of the patients in need receives targeted interventions [8].

Routine QOL assessment can contribute to optimized symptom management by eliciting unrecognized problems while monitoring the course of patients' level of functioning and well-being. Even more so as self-reports are reported to be more sensitive to underlying changes in functional status and are reported sooner than rated by physicians [48]. To support feasibility in clinical routine, QOL assessment can be done computer-based, allowing easy data processing and real-time feedback to the medical staff [49]. Furthermore, the electronic approach allows building large datasets which can be used to calculate specific reference values. Software providing these features is currently implemented in oncological in- and outpatients units in several hospitals in Austria [50-52].

## Conclusion

The present study underlines the importance of longitudinal assessment of QOL in order to facilitate the identification of symptom burden in OC patients over the course of treatment. We found that patients show high levels of fatigue, anxiety and depressive symptoms and severely impaired QOL post-surgery (i.e. at start of chemotherapy) but their condition improves considerably throughout chemotherapy reaching nearly general population symptoms levels until aftercare. To support medical as well as psycho-oncological symptom management, routinely conducted QOL monitoring is recommended. In future research, especially the course of QOL and psychosocial issues for long-term survivors of OC require further investigation.

## Competing interests

The authors declare that they have no competing interests.

## Authors' contributions

VM, AG, AGZ, BSU and BH conceived of the study and participated in its design and coordination. AG and PW carried out the assessments. EMG and JMG participated in the design of the study and performed the statistical analysis. VM, EMG, PW and MS were engaged in manuscript writing. All authors read and approved the final manuscript.

## Pre-publication history

The pre-publication history for this paper can be accessed here:

http://www.biomedcentral.com/1471-2407/12/77/prepub
